# Association between Maternal Serum 25-Hydroxyvitamin D Concentrations and the Risk of Preterm Birth in Central Sudan: A Case–Control Study

**DOI:** 10.3390/nu14040891

**Published:** 2022-02-20

**Authors:** Somia K. Abdelrahiem, Manal E. Sharif, Nadiah ALhabardi, Osama Al-Wutayd, Ishag Adam

**Affiliations:** 1 Department of Obstetrics and Gynecology, University of Gezira, Sudan. P.O. Box 20, Wad Medani 21111, Sudan; soumia@uofg.edu.sd; 2 College of Medicine, King Khalid University, Abha 61421, Saudi Arabia; mnoor@kku.edu.sa; 3 Department of Obstetrics and Gynecology, Unaizah College of Medicine and Medical Sciences, Qassim University, Unaizah 56219, Saudi Arabia; n.alhabrdi@qu.edu.sa (N.A.); ia.ahmed@qu.edu.sa (I.A.); 4 Department of Family and Community Medicine, Unaizah College of Medicine and Medical Sciences, Qassim University, Unaizah 56219, Saudi Arabia

**Keywords:** preterm birth, pregnancy, vitamin, 25(OH)D, Sudan

## Abstract

There are few published studies on the association between vitamin D concentrations and preterm birth (PB) in sub-Saharan Africa. The current study aimed to assess the association between 25-hydroxyvitamin D (25[OH)] D) levels and PB. A matched case–control study (60 women in each arm) was conducted in Medani maternity hospital in central Sudan. The cases were women with spontaneous PB, and healthy women with term deliveries were the controls. The clinical/medical and obstetric history was gathered using a questionnaire. The enzyme-linked immunosorbent assay was used to measure the serum 25(OH)D levels. Women with PB had significantly lower median (interquartile range) 25(OH)D concentrations compared with the controls (18.4 (7.3) ng/mL vs. 20.2 (16.5) ng/mL, *p* = 0.001). Forty-two (70.0%) women with PB and 29 (48.3%) women in the control group had vitamin D deficiency (25(OH)D level ≤ 20 ng/mL). The results of the multivariable logistic regression showed that the 25(OH)D concentrations were negatively associated with PB (adjusted odds ratio (aOR) = 0.92, 95% confidence interval (CI) = 0.87–0.97). Vitamin D-deficient pregnant women were at a higher risk of PB (aOR = 2.69, 95% CI = 1.17–6.23). Low 25(OH)D concentrations were found at the time the variable was determined in women with spontaneous PB and were an independent risk factor for PB.

## 1. Introduction

Preterm birth (PB) is defined as the birth of the baby before 37 completed weeks of gestation or 259 days, which is calculated from the final day of the last menstrual period [1]. PB is a big health problem worldwide, as it has been estimated that 4.84 million PBs occurred in 2014, and the majority of all PBs (three quarters) were reported in Africa and South Asia [2]. PB is a major cause of several adverse outcomes such as perinatal death [3] and neonatal death [4]. Moreover, PB also has an economic burden, as these newborn might require admission to neonatal intensive care units [5]. It has recently been reported that sub-Saharan African countries have high rate of PB—for example, 13.3% and 18.3% was the prevalence of PB in Ethiopia and in Kenya, respectively [6,7]. Vitamin D, a fat-soluble vitamin, has a major role in the homeostasis of calcium, primary bone health, as well as cell proliferation and immune function [8,9]. Previous reports have shown that several maternal and perinatal adverse effects such as gestational diabetes, preeclampsia, PB and small for gestation age and intrauterine growth restriction are associated with low vitamin D levels in pregnancy [10,11]. Previous studies on the associations between vitamin D and PB showed inconclusive results—while some of the studies showed that serum vitamin D levels are lower in women with PB and reported that vitamin D deficiency is associated with an increased risk of PB [10,11,12,13,14,15,16], other studies did not identify an association between low serum 25(OH)D concentrations/vitamin D deficiency and the risk of PB [17,18,19,20,21]. Moreover, most of these studies were conducted in countries outside Africa, with few published data on the association between serum 25(OH)D concentrations/vitamin D deficiency and the risk of PB in Africa [13,14]. As with many other diseases, the aetiology and pathophysiology of PB might differ in a different setting—for example, in the case of PB with a communicable disease such as malaria [22]. There are no published data on the association between serum 25(OH)D concentrations/vitamin D deficiency and risk of PB in Sudan. We recently reported that the majority (94%) of pregnant Sudanese women have vitamin D deficiency (≤20 ng/mL) [23]. Thus, it is vital to determine if vitamin D levels are associated with PB in Sudan. This will generate the data necessary for evidence-based interventions (e.g., recommending the administration of vitamin D during pregnancy, especially for the high-risk group of women). Therefore, the current study was conducted to investigate the serum 25(OH)D levels in women with PB.

## 2. Subjects and Methods

### 2.1. Study Setting and Design

A matched (age and gestational age) case–control study was conducted at Medani Maternity Hospital in Gezira, Sudan, from June to December 2020.

### 2.2. Case and Control Definition

The cases were pregnant women who presented with spontaneous PB during this time. A healthy pregnant woman matched for age and gestational age without systemic disease, such as diabetes mellitus, thyroid disease, hypertension, renal disease, and proteinuria, served as the control. Smokers, women with multiple pregnancies, diabetic women and participants whose foetuses had major anomalies or died were excluded from the study. None of the women in the cases or in the controls were on vitamin D supplements. Controls were followed up until they reached term (37 weeks), otherwise they were not considered.

After providing informed written consent (included blood sampling), the participants were asked about their sociodemographic, obstetric and clinical data (age, parity, educational level, antenatal attendance and history of miscarriage). Weight and height were measured using standard procedures to compute body mass index (BMI), and haemoglobin concentration was measured at the time of the delivery using an automated haematology analyser and following the manufacturer’s instructions (Sysmex KX-21, Osaka, Japan).

### 2.3. Processing of Blood Sample

Five millilitres of blood was taken from the cubital vein into a plain tube, and it was allowed to clot at room temperature. The blood was then centrifuged and stored at −20 °C until we performed the assay of 25(OH)D by using the enzyme-linked immunosorbent assay (fully automaTable 450 nm, reference wavelength between 620 and 650 nm) following the manufacturer’s instructions (Euroimmun, Lubeck, Germany). Manufacturer quality control measures and 6 levels of standard solutions (calibrator) set between 0 and 120 ng/mL were applied for each assay. 

### 2.4. Sample Size Calculation

The sample was (60 women in each arm) calculated as the ratio of 1:1 between the cases and the controls. Depending on our previous reports on the serum concentration of 25(OH)D (15.0 ng/mL) among pregnant Sudanese women [23], we assumed that women with PB would have a concentration of 25(OH)D of 10.0 ng/mL. Based on our recent reports on the level of 25(OH)D among women with preeclampsia [24], we assumed a mean difference of 5 ng/mL in the serum 25(OH)D concentration between the women who had PB and the healthy controls. This sample size (60 women in each arm) was used to achieve 80% power with precision of 5%.

### 2.5. Data Analysis

Statistical analysis was performed using Statistical Package for the Social Sciences^®^ (SPSS^®^) IBM SPSS Statistics for Windows, version 22.0 (SPSS Inc., New York, United States). The proportions of the studied variables were expressed as frequency (%). The continuous data, including 25(OH)D concentration, were evaluated for normality using the Shapiro–Wilk test and were expressed as means (standard deviation) or median (interquartile range (IQ)) as applicable. Associations between specific variables, such as age, parity, educational level, occupation, history of miscarriage, BMI and 25(OH)D concentration, and PB were assessed using bivariate analysis. Variables with a *p*-value of ≤ 0.20 were selected for the construction of multivariable models that considered crude associations between PB and the variables. Backward elimination (conditional) was performed to adjust the model for covariates. Adjusted odds ratios (aORs) and 95% confidence intervals (CIs) were computed. A two-sided *p*-value of <0.050 was considered to be statistically significant.

## 3. Results

There was no significant difference in the age, educational level, employment, haemoglobin level, and BMI between women with PB (*n* = 60) and the controls (*n* = 60). Compared with the controls, a greater number of women with PB had low parity, attended antenatal care and had a history of miscarriage. Forty-two (70.0%) women with PB and 29 (48.3%) women in the control group had vitamin D deficiency (25(OH)D level ≤ 20 ng/mL), as detailed in Table 1.

**Figure 1:**
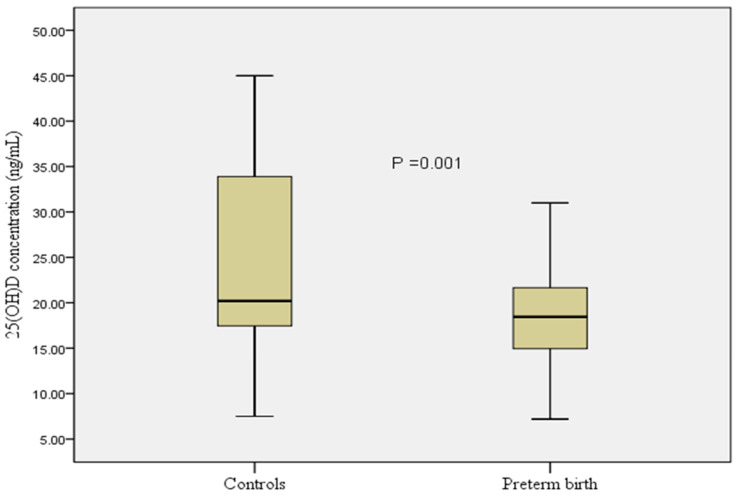
Comparing serum 25(OH)D concentration between women with preterm birth and the controls.

The median (IQ) for the serum 25(OH)D concentration was substantially lower in women with PB than in the controls (18.4 (7.3) ng/mL vs. 20.2 (16.5) ng/mL, respectively) (Figure 1).

The results of the multivariable logistic regression show that the 25(OH)D concentrations were negatively associated with PB (aOR = 0.92, 95% CI = 0.87–0.97). Vitamin D-deficient pregnant women were at a higher risk of PB (aOR = 2.69, 95% CI = 1.17–6.23). Moreover, antenatal care level and a history of miscarriage/PB were associated with PB. Parity and educational level were associated with PB in the non-adjusted model (detailed in Table 2).

## 4. Discussion

In the current study, the serum 25(OH)D level was inversely associated with PB. The risk of PB decreased by 8.0% for each nanogram-per-millilitre increase in serum 25(OH)D level (aOR = 0.92). In addition, vitamin D-deficient women had a 2.6-times greater risk of PB.

Our result agrees with the results (serum 25(OH)D concentrations/vitamin D deficiency and the risk of PB) of previous studies which were recently conducted in sub-Saharan Africa, namely in Ghana [13] and in Nigeria [14]. Several previous studies have shown that the serum vitamin D levels in women with PB were significantly lower than their peer controls and reported that vitamin D deficiency during pregnancy is associated with an increased risk of PB [10,11,12,15,16]. In a previous meta-analysis (2013) which included 24 studies, Wei et al. reported that a low maternal vitamin D level was associated with an increased risk of PB (OR = 1.58) [10]. Later on (2016), Qin et al. [16], in their meta-analysis which included 10 studies and enrolled 10,098 pregnant women, reported that pregnant women with vitamin D deficiency were at higher risk to have PTB (OR = 1.29). One year later (2017), Zhou [25] et al., in their meta-analysis which included 24 articles (18 were observational studies and six were randomized clinical trials), concluded that maternal 25-OHD deficiency was associated with an increased risk of PB (OR = 1.25). On the other hand, several previous studies did not identify an association between low serum 25(OH)D concentrations/vitamin D deficiency and the risk of PB [18,19,20,21]. Recently, Lian et al., in their meta-analysis which included 24 studies, concluded that vitamin D deficiency in early and late pregnancy was not associated with increased risk of PB; however, they reported that vitamin D deficiency in the second trimester of pregnancy was associated with increased risk of PB [17]. It is worth mentioning that a recent meta-analysis which included 55 studies showed that only a relatively high (<75 nmol/L) cut-off level of vitamin D was associated with PB [26]. It is worth mentioning that the difference in the results of these studies might be explained by the difference in the sociodemographic characteristics, the difference in the prevalence of vitamin D deficiency and its associated factors in the differ settings, and the difference in the cut-off points used to identify vitamin D deficiency. The immunosuppressive impact of vitamin D on proinflammatory cytokine concentrations has been observed [27,28]. Vitamin D has been observed to reduce the oxidative stress that results in placental trophoblast invasion [29]. Spontaneous PB is commonly attributed to infection and its associated inflammation [30]. Vitamin D plays an important role as an immune modulator, and it can reduce the possibility of PB, inhibiting inflammation by means of its antimicrobial activity [31]. A previous report showed that vitamin D might affect acquired as well as innate immunity, which is an important factor at the foetal–maternal interface [32]. Moreover, vitamin D has a function as an intracrine regulator of and has an important role in the activation of innate immune responses in the placental tissue [33,34]. Interestingly, vitamin D can reduce bacterial infections such as bacterial vaginosis through the induction of cathelicidin in several tissues of the maternal and foetal parts of the placenta. [35]. Thus, vitamin D might reduce the risk of PB by decreasing placental infection/colonization by these organism (bacterial vaginosis) [30,36]. Although we mainly conducted this study to assess vitamin D level and its associations with PB, our study shows that a lack of antenatal care and a history of miscarriage/PB were associated with PB. This agrees with the findings of previous studies in other African countries [6,7]. Generally, there is a high rate of vitamin D deficiency in sunbathed countries such as Sudan, which could be explained by skin pigmentation as well as nutritional factors [37].

## 5. Limitations

We measured the 25-OHD levels at the time of delivery, meaning that these data cannot not reflect the whole picture throughout the course of the pregnancy. A longitudinal study that assesses the 25-OHD levels in early and mid pregnancy as well as in the later weeks of gestation is needed. Several factors such as infection of the urinary tract or cervical/uterine infections, and nutrients including other vitamins such as folic acid and zinc which might have their own effect on PB were not measured. Inflammatory factors such c-reactive protein and cytokines were not measured too. Consequently, larger longitudinal studies are needed to provide more evidence so as to prove this finding.

## 6. Conclusions

Low 25(OH)D concentrations were found at the time the variable was determined in women with spontaneous PB and were an independent risk factor for PB.

## Figures and Tables

**Table 1:** Univariate and bivariate analysis of sociodemographic and clinical variables in women with preterm birth and the controls.

Variables	Preterm Birth (*n* = 60)	Controls (*n* = 60)	OR (95% CI)	*p*-Value
	Median (Interquartile Range)			
Age (years)	28.5 (9.5)	28.5 (4.7)	0.98 (0.92–1.05)	0.732
Parity	2.0 (3.0)	3.0 (3.0)	0.80 (0.64–0.99)	0.047
BMI (kg/m^2^)	25.4 (6.1)	26.1 (5.0)	0.96 (0.89–1.04)	0.414
Haemoglobin level (g/dl)	11.7 (1.7)	11.8 (1.5)	1.01(0.80–1.26)	0.953
25(OH)D concentration (ng/mL)	18.4 (7.3)	20.2 (16.5)	0.92 (0.87–0.96)	0.001
Frequency (proportion)				
Education level				
Secondary or higher	15 (25.0)	22 (36.7)	Reference	
Primary or lower	45 (75.0)	38 (63.7)	1.73 (0.79–3.81)	0.168
Antenatal care				
≥2 visits	51 (85.0)	59 (98.3)	Reference	
˂2 visits	9 (15.0)	1 (1.7)	4.07 (1.55–10.54)	0.004
Employment				
Housewives	55 (91.7)	54 (90.0)	Reference	
Employed	5 (8.3)	6 (10.0)	0.81 (0.23–2.84)	0.752
History of miscarriage				
No	49 (81.7)	57 (95.0)	Reference	
Yes	11 (18.3)	3 (5.0)	4.26 (1.12–16.16)	0.033
Vitamin D deficiency				
No	18 (30.0)	31 (51.7)	Reference	0.017
Yes	42 (70.0)	29 (48.3)	2.49 (1.17–5.27)	

25(OH)D: 25-hydroxyvitamin D; BMI: body mass index; CI: confidence interval; OR: odds ratio.

**Table 2:** Multivariable analysis of the non-adjusted and adjusted odds ratio of the factors associated with preterm birth (cases) compared with the controls.

	Non-Adjusted		Adjusted	
Variables	OR (95% CI)	*p*-Value	aOR (95% CI)	*p*-Value
Parity	0.79 (0.61–1.01)	0.064		
25-hydroxyvitamin D concentration (ng/mL) *	0.92 (0.87–0.97)	0.006	0.92 (0.87–0.97)	0.006
Education level				
Secondary or higher	Reference			
Primary or lower	2.21 (0.89–5.43)	0.084		
Antenatal care				
≥2 visits	Reference		Reference	
˂2 visits	4.78 (1.69–13.49)	0.003	4.78 (1.69–13.49)	0.003
History of miscarriage				
No	Reference		Reference	
Yes	4.86 (1.14–20.62)	0.032	4.86 (1.14–20.62)	0.032
Vitamin D deficiency *				
No	Reference		Reference	
Yes	2.84 (1.21–6.64)	0.016	2.69 (1.17–6.23)	0.012

CI: confidence interval; OR: odds ratio. * These were entered one by one (one of them in each model). History of miscarriage and 25-hydroxyvitamin D concentration (ng/mL) were the model fit variables.

## Data Availability

The data presented in this study are available on request from the last author.

## References

[1] (2012). Born too Soon: The Global Action Report on Preterm Birth. Australas. Med. J..

[2] Chawanpaiboon S., Vogel J.P., Moller A.B., Lumbiganon P., Petzold M., Hogan D., Landoulsi S., Jampathong N., Kongwattanakul K., Laopaiboon M. (2019). Global, regional, and national estimates of levels of preterm birth in 2014: A systematic review and modelling analysis. Lancet Glob. Health.

[3] Mondal M.N.I., Hossain M.K., Ali M.K. (2009). Factors influencing infant and child mortality: A case study of Rajshahi District, Bangladesh. J. Hum. Ecol..

[4] Goldenberg R.L., Culhane J.F., Iams J.D., Romero R. (2008). Epidemiology and causes of preterm birth. Lancet.

[5] Zainal H., Dahlui M., Soelar S.A., Su T.T. (2019). Cost of preterm birth during initial hospitalization: A care provider’s perspective. PLoS ONE.

[6] Aregawi G., Assefa N., Mesfin F., Tekulu F., Adhena T., Mulugeta M., Gebreayezgi G. (2019). Preterm births and associated factors among mothers who gave birth in Axum and Adwa Town public hospitals, Northern Ethiopia, 2018. BMC Res. Notes.

[7] Wagura P.M., Wasunna A., Laving A., Wamalwa D., Ng’Ang’A P. (2018). Prevalence and factors associated with preterm birth at kenyatta national hospital. BMC Pregnancy Childbirth.

[8] Wacker M., Holiack M.F. (2013). Vitamin D-effects on skeletal and extraskeletal health and the need for supplementation. Nutrients.

[9] Holick M.F. (2012). Evidence-based D-bate on health benefits of vitamin D revisited. Derm. Endocrinol..

[10] Wei S.Q., Qi H.P., Luo Z.C., Fraser W.D. (2013). Maternal vitamin D status and adverse pregnancy outcomes: A systematic review and meta-analysis. J. Matern. Neonatal Med..

[11] Miliku K., Vinkhuyzen A., Blanken L.M.E., McGrath J.J., Eyles D.W., Burne T.H., Hofman A., Tiemeier H., Steegers E.A.P., Gaillard R. (2016). Maternal vitamin D concentrations during pregnancy, fetal growth patterns, and risks of adverse birth outcomes. Am. J. Clin. Nutr..

[12] María Pérez-Castillo Í., Rivero-Blanco T., Alejandra León-Ríos X., Expósito-Ruiz M., Setefilla López-Criado M., Aguilar-Cordero M.J. (2020). Associations of Vitamin D Deficiency, Parathyroid hormone, Calcium, and Phosphorus with Perinatal Adverse Outcomes. A Prospective Cohort Study. Nutrients.

[13] Fondjo L.A., Tashie W., Owiredu W.K.B.A., Adu-Gyamfi E.A., Seidu L. (2021). High prevalence of vitamin D deficiency among normotensive and hypertensive pregnant women in Ghana. BMC Pregnancy Childbirth.

[14] Oluwole A.A., Okunade K.S., Okojie O.E. (2019). Maternal serum vitamin D levels and preterm delivery among low-risk parturients in Lagos, Nigeria. Int. J. Gynecol. Obstet..

[15] Jao J., Freimanis L., Mussi-Pinhata M.M., Cohen R.A., Monteiro J.P., Cruz M.L., Branch A., Sperling R.S., Siberry G.K. (2017). Severe Vitamin D Deficiency in Human Immunodeficiency Virus-Infected Pregnant Women is Associated with Preterm Birth. Am. J. Perinatol..

[16] Qin L.L., Lu F.G., Yang S.H., Xu H.L., Luo B.A. (2016). Does maternal Vitamin D deficiency increase the risk of preterm birth: A meta-analysis of observational studies. Nutrients.

[17] Lian R.H., Qi P.A., Yuan T., Yan P.J., Qiu W.W., Wei Y., Hu Y.G., Yang K.H., Yi B. (2021). Systematic review and meta-analysis of vitamin D deficiency in different pregnancy on preterm birth: Deficiency in middle pregnancy might be at risk. Medicine.

[18] Pérez-López F.R., Pasupuleti V., Mezones-Holguin E., Benites-Zapata V.A., Thota P., Deshpande A., Hernandez A.V. (2015). Effect of vitamin D supplementation during pregnancy on maternal and neonatal outcomes: A systematic review and meta-analysis of randomized controlled trials. Fertil. Steril..

[19] Yu L., Guo Y., Ke H.J., He Y.-S., Che D., Wu J.L. (2019). Vitamin D status in pregnant women in southern China and risk of preterm birth: A large-scale retrospective cohort study. Med. Sci. Monit..

[20] Vivanti A.J., Monier I., Salakos E., Elie C., Tsatsaris V., Senat M.V., Jani J., Jouannic J.M., Winer N., Zeitlin J. (2020). Vitamin D and pregnancy outcomes: Overall results of the FEPED study. J. Gynecol. Obstet. Hum. Reprod..

[21] Monier I., Baptiste A., Tsatsaris V., Senat M.V., Jani J., Jouannic J.M., Winer N., Elie C., Souberbielle J.C., Zeitlin J. (2019). First Trimester Maternal Vitamin D Status and Risks of Preterm Birth and Small-For-Gestational Age. Nutrients.

[22] Mahamar A., Andemel N., Swihart B., Sidibe Y., Gaoussou S., Barry A., Traore M., Attaher O., Dembele A.B., Diarra B.S. (2021). Malaria Infection Is Common and Associated with Perinatal Mortality and Preterm Delivery Despite Widespread Use of Chemoprevention in Mali: An Observational Study 2010 to 2014. Clin. Infect. Dis..

[23] Mahmoud S.Z., Saad A.A., Mohieldein A.H., Nasr A.M., Adam I. (2019). Serum level of 25-hydroxyvitamin D and obesity among early pregnant women. J. Obstet. Gynaecol. Res..

[24] Abdelrahiem S.K., Ahmed A.B.A., Sharif M.E., Adam I. (2021). Association between maternal serum 25-hydroxyvitamin D concentrations and the risk of pre-eclampsia in central Sudan: A case-control study. Trans. R. Soc. Trop. Med. Hyg..

[25] Zhou S.S., Tao Y.H., Huang K., Zhu B.B., Tao F.B. (2017). Vitamin D and risk of preterm birth: Up-to-date meta-analysis of randomized controlled trials and observational studies. J. Obstet. Gynaecol. Res..

[26] Aguilar-Cordero M.J., Lasserrot-Cuadrado A., Mur-Villar N., León-Ríos X.A., Rivero-Blanco T., Pérez-Castillo I.M. (2020). Vitamin D, preeclampsia and prematurity: A systematic review and meta-analysis of observational and interventional studies. Midwifery.

[27] de Souza E.A., Pisani L.P. (2020). The relationship among vitamin D, TLR4 pathway and preeclampsia. Mol. Biol. Rep..

[28] Smith T.A., Kirkpatrick D.R., Kovilam O., Agrawal D.K. (2015). Immunomodulatory role of Vitamin D in the pathogenesis of preeclampsia. Expert Rev. Clin. Immunol..

[29] Xu J., Jia X., Gu Y., Lewis D.F., Gu X., Wang Y. (2017). Vitamin D reduces oxidative stress-induced procaspase-3/ROCK1 activation and mp release by placental trophoblasts. J. Clin. Endocrinol. Metab..

[30] Grant W.B. (2011). Adequate vitamin D during pregnancy reduces the risk of premature birth by reducing placental colonization by bacterial vaginosis species. MBio.

[31] Liu N.Q., Hewison M. (2012). Vitamin D, the placenta and pregnancy. Arch. Biochem. Biophys..

[32] Liu N.Q., Kaplan A.T., Lagishetty V., Ouyang Y.B., Ouyang Y., Simmons C.F., Equils O., Hewison M. (2011). Vitamin D and the regulation of placental inflammation. J. Immunol..

[33] Liu N., Kaplan A.T., Low J., Nguyen L., Liu G.Y., Equils O., Hewison M. (2009). Vitamin D induces innate antibacterial responses in human trophoblasts via an intracrine pathway. Biol. Reprod..

[34] Evans K.N., Nguyen L., Chan J., Innes B.A., Bulmer J.N., Kilby M.D., Hewison M. (2006). Effects of 25-hydroxyvitamin D3 and 1,25-dihydroxyvitamin D3 on cytokine production by human decidual cells. Biol. Reprod..

[35] Dunlop A.L., Taylor R.N., Tangpricha V., Fortunato S., Menon R. (2011). Maternal vitamin D, folate, and polyunsaturated fatty acid status and bacterial vaginosis during pregnancy. Infect. Dis. Obstet. Gynecol..

[36] Fichorova R.N., Onderdonk A.B., Yamamoto H., Delaney M.L., DuBois A.M., Allred E., Levitonc A. (2011). Maternal microbe-specific modulation of inflammatory response in extremely low-gestational-age newborns. MBio.

[37] Gaffer A.A., Rayis D.A., Elhussein O.G., Adam I. (2019). Vitamin D status in Sudanese pregnant women: A cross-sectional study. Trans. R. Soc. Trop. Med. Hyg..

